# Is modernization widening cultural differences?

**DOI:** 10.1093/pnasnexus/pgag021

**Published:** 2026-03-10

**Authors:** Thomas Talhelm

**Affiliations:** University of Chicago Booth School of Business, Chicago, IL, USA

**Keywords:** modernization, globalization, capitalism, free trade, values, culture, culture change, China, rice theory

## Abstract

Some researchers have argued that modernization is making cultures more individualistic and shrinking cultural differences. Here, I points to counterintuitive new evidence that modernization is increasing some cultural differences. For example, across the 40 years of the World Values Survey, nations are becoming more different in their values, rather than more similar. I propose that modernization sometimes acts like water on a seed, giving people resources that they decide to use in ways that depend on their pre-existing values and beliefs. The seed model offers a framework for understanding findings that do not fit classic modernization theory from domains of psychology to behavioral genetics. I outline critical questions that this model needs to answer if it is to become useful, such as where the seeds of culture come from and when we should predict decreasing versus increasing differences as a result of modernization.

A popular idea is that modernization reduces cultural differences. The basic argument is that “nations become more culturally similar as they experience economic growth” and global income inequality declines (1, 2). Back in 1842, Marx and Engels already believed differences were shrinking when they published *The Communist Manifesto.* They wrote that “National differences and antagonisms between peoples are daily more and more vanishing, owing to the development of the bourgeoisie, to freedom of commerce, to the world-market” (3). Yet some recent evidence is pointing to the idea that modernization may actually be increasing some cultural differences.

## Evidence for the loss of cultural differences

Economic growth is a central element of modernization, but the broader concept also includes shifts toward more technology, social exchange, education, and urbanization (4, 5). Scholars have argued about whether the world is becoming better off over the last 100 years (6), but it is clear that the world has become more modernized in terms of components like education and urbanization (7, 8).

Along with that modernization, there is compelling evidence for the loss of some kinds of cultural differences. One clear example is that the world is quickly losing languages (and presumably cultural differences with it). According to one estimate, a language goes extinct every 10 days (9).

There is also strong evidence in the domains of marriage and childbirth. Modernization seems to consistently increase divorce rates and decrease birth rates (10). One researcher described the link between modernization and birth rates as, “one of the most solidly established and generally accepted empirical regularities in the social sciences” (11).

To be fair, “consistent” does not mean “always.” For example, the divorce rate in the United States has declined recently (12). There is also some evidence that birth rates decline with economic development but might go back up at very high levels of development (11). However, the overall pattern is strong.

Researchers can look at this pattern repeated in different cultures and conclude that modernization has the same effect from Singapore to Santiago. In line with that view, the political scientist Ronald Inglehart wrote that “economic development tends to push societies in a common direction” (5). People who look at the changes in birth rates could logically conclude that the effect of modernization is predictable. All we need to know is whether a society is modernizing and we can predict what is happening to birth rates there. We do not need to know anything about the people there to predict the consequences of modernization.

## Modernization as radiation

In people's beliefs about modernization, we can draw a parallel to radiation. Radiation from things like x-ray machines and nuclear reactions weakens the human body, similar to how modernization may weaken traditional culture. I think the parallel to radiation can be helpful because it can illustrate tacit assumptions underlying how people think about modernization:

1. Modernization is an outside force that can work its way inside us.
2. Modernization acts on people regardless of their values or beliefs. An alternative version is that modernization does depend on people’s values, but modernization produces the same outcomes around the world because people all want the same things, such as freedom and choice.
3. The effect of modernization is predictable and reliable. One slight divergence between radiation and modernization is that radiation has an element of probability, especially at low doses. Low doses sometimes cause cancer and sometimes do not.

One of those supposedly reliable effects is individualism. Some researchers have argued that modernization causes value shifts toward individualism so reliably that these effects “are not culture specific but universal” (13). For example, researchers cataloged markers like divorce and childbirth around the world and concluded that individualism is rising in most countries (14). If most of the world is moving toward individualism, this could cause convergence. If modernization works like radiation and produces a predictable effect, it is logical to predict that modernization will diminish cultural differences.

## Evidence for growing cultural differences

However, provocative recent findings are pointing to widening cultural differences. In one study, researchers analyzed cultural differences in the World Values Survey from its inception in 1981 (15). The survey asked people to rate the importance of a range of values, such as teaching children “obedience” and “responsibility.” They estimated cultural differences by calculating the standard deviation across 40 different value questions.

**Fig. 1.**
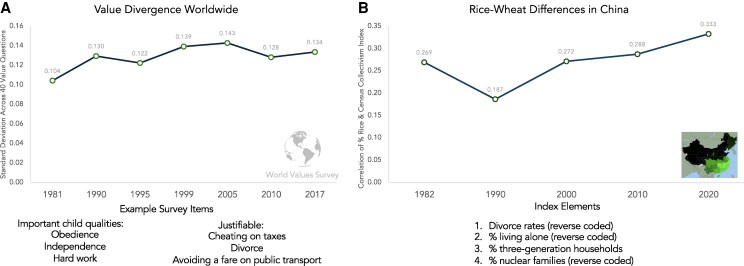
Examples of increasing cultural differences worldwide and in China. (A) The standard deviation between countries on 40 value questions from the World Values Survey (15). (B) The correlation between Chinese provinces' historical rice farming and collectivism, as measured by census indicators such as living in three-generation households versus living alone.

From 1981 to 2022, values across countries became more different, not more similar (Fig. 1). Variation in the obedience question rose 42% over those 40 years. Across all 40 values, variation rose 28%.

One complication is that the World Values Survey has included more countries over time. That means that the sample has changed since the start of the survey. However, the pattern of divergence remained after limiting the sample to countries that had participated in the same waves over time.

Another complication with the World Values Survey is that it is difficult to infer causality by comparing differences across countries. Countries differ in many ways, and that can make it hard to rule out alternative explanations. Comparisons within countries can offer cleaner (albeit still imperfect) testing grounds.

China offers an interesting testing ground because it has modernized so deeply over the last 40 years, yet it has cultural differences linked to regions' histories of rice farming versus wheat farming (16). Paddy rice is built on irrigation networks, which forced farmers to coordinate flooding, draining, dredging, and repair (17). Rice also required more labor than crops like wheat. Rice villages coped by developing customs for exchanging labor. Today, these legacies help explain why China's rice farming regions are more collectivistic than the wheat farming regions (16, 18).

If differences in China come from in regions' history of farming, it is easy to predict that these differences should be decreasing with modernization. We can test this prediction by measuring differences over time in Census data. Researchers have used Census indicators to estimate collectivism (19). Collectivistic areas in the United States, Japan, China, and around the world tend to have lower divorce rates, fewer people living alone, and more three-generation households (16, 19).

Modern Census data are available for Chinese provinces from 1982 to 2020 [more details on the collectivism index for China are in Wei et al. (20)]. During that time period, China modernized dramatically. Gross domestic product (GDP) per capita grew from approximately US$100 to US$10,000. It would be difficult to find a corner of the world that catapulted farther along the trajectory of modernization during those decades.

To measure regions' long-run history of rice farming, I used statistics on how much cultivated land each province devoted to rice farming (16). My goal with these land statistics is to estimate the cultural history of rice farming across regions, not the year-to-year changes that happened in between the censuses recently. These numbers are my estimate of the seed.

Despite the rapid modernization, the correlation between rice farming and this basket of collectivism indicators actually grew (Fig. 1). In 1982, the correlation was not significant (*r* = 0.18, *P* = 0.152). In 2020, it was (*r* = 0.38, *P* = 0.034). At least by this metric, rice-wheat differences in China are growing with modernization.

That is all the more interesting because rice and wheat provinces started from nearly identical economic baselines. In 1982, rice provinces started with an average GDP per capita of 708 Yuan, compared with 613 Yuan in wheat provinces (for summary statistics like this, I am following previous research by comparing rice provinces [>50% cultivated land devoted to rice] and wheat provinces [<50%]) (16). I suspect that difference is too small to mean anything for culture. But if it did, rice areas should have started out lower on the collectivism index. Yet, the census statistics from 1982 show no trend in that direction (*r* = 0.18, *P* = 0.152).

From that starting point, rice and wheat areas both became more modernized. But a skeptical reader could ask whether the wheat areas become more modernized. If so, the widening of rice-wheat cultural differences could just be because modernization happens to be more concentrated in the region that was more individualistic to begin with. This question makes it tricky to study the effect of the Industrial Revolution on individualism because some people have argued that economic growth causes individualism (14), while other people have argued that the West became wealthy at least partly because it was already individualistic to begin with (21).

Again, the rice and wheat areas of China provide a convenient case because that confounding is absent. Both areas experienced wild economic growth between 1982 and 2020, but rice areas grew more (11,755%) than wheat areas (10,139%). If growth leads to individualism, rice areas should have moved more toward individualism than wheat areas, which would cause them to converge. Yet the opposite happened.

To be fair, researchers in one study argued that China is an exception to modernization theory (14). They analyzed 5 questions potentially related to individualism from the World Values Survey, such as agreeing with the goal of “Giving people more say in important government decisions.” They analyzed 53 countries and found that China was one of only 5 that showed a decrease over time.

However, the World Values Survey study in Fig. 1 found divergence over a broader time scale (15). The study in Fig. 1 used all value questions from the World Values Survey over more time waves of the survey. The fact that the larger study found divergence gives at least some evidence that cultural divergence is not just a China phenomenon (15).

## Modernization might be water on a seed

Why are some cultural differences increasing with modernization? I want to offer the possibility that modernization sometimes acts like water on a seed. The seed is some pre-existing difference between people. It could be a cultural value or belief. The effect of modernization is like water on that seed, helping those values grow. If different cultures start with different seeds, then modernization will cause those differences to grow. The analogy to a growing plant offers a qualitatively different model from radiation:

1. The effect of modernization is a combination of outside forces and forces inside people's heads.
2. People decide how they use the resources that modernization provides.
3. The effect of modernization differs depending on the values and beliefs in people's heads.

The seed model could explain divergence in the World Values Survey data. In that data, it is particularly interesting to look at non-Western cultures that became much wealthier over time because they might have different seeds from Western cultures. For example, in Hong Kong, the percentage of people saying it is important to teach children responsibility went up from about 19% to 52% (15). In Canada, it went down slightly (6%), even though GDP per-capita growth was similar to Hong Kong over that period of time.

Maybe Hong Kong or that particular survey question are exceptions? The data from Asia and North America as a whole suggest not. The divergence between Canada and Hong Kong parallels the divergence between North America and Asia from the first wave to the seventh wave of the World Values Survey [see Figure 2 from Jackson and Medvedev (15)].

## Alternatives to the idea that modernization homogenizes culture

The seed model has roots in earlier ideas. The closest parallel is probably in the writings of the French historian Fernand Braudel. In 1969, he wrote that the poverty of the Middle Ages made it so that “all civilizations were thus deployed within a very narrow range of possibilities” (22). Poverty created “a profound similarity through time and space,” he argued. In his vision, modernization freed people from the limitations of poverty, and differences between civilizations grew.

Another person who denied convergence was Samuel Huntington in his influential book *The Clash of Civilizations the Remaking of World Order* (23). Huntington argued that conflicts in the modern world are rooted in long-lasting differences between civilizations. He wrote that people had overestimated how much modernization would push the world toward a shared set of values.

One of his claims that caught readers' eyes was that “the world is becoming more modern and less Western” (23, 24). The reason that this raised eyebrows is that it conflicted with the popular “end of history” idea popularized by Francis Fukuyama (Fukuyama was Huntington's PhD student) (25). History can “end” if some forms of government and economic systems are more efficient and lead to better outcomes than others. Over time, cultures will converge on the more efficient forms of liberal democracy and market economies as “the final form of human government” (25).

Fukuyama's idea makes sense if we think that civilizations follow a path of progress over time. Fukuyama did not argue that the end of history meant the end of cultural differences (26). But if people believe that political institutions and economic systems shape culture, it would be logical to take that to mean cultural differences will shrink as more and more societies converge on the same economic and political systems.

However, another view is that cultures could adopt similar institutions but shape them in their own cultural way. This is the idea behind “multiple modernities,” from the sociologist Shmuel Eisenstadt (27). Take universities in Japan and the United States as examples. Japan developed modern universities, just like the United States. Japanese universities have laboratories, PhD students, and tenure systems. On the surface, it could be easy to conclude that these two cultures have converged in their higher education systems.

But deeper differences remain. One difference is what three scholars in Japan described as “academic inbreeding” (28). Inbreeding happens when universities hire their own PhD students as professors, and they argued inbreeding rates are high in Japan. This happens in the United States too, but rates tend to be lower (29). On top of that, pay in Japan often depends more on seniority than in other cultures (30). When pay is based on how long people have been with an organization, that disincentivizes people from switching jobs.

Less switching and more inbreeding maps onto larger cultural differences in “relational mobility” (31). Relationally mobile cultures have more opportunities to meet people, more freedom to break relationships, and more choice in who to be with. In a study of 39 cultures around the world, people in Japan reported the fewest opportunities to meet new people and the least choice in their social relationships (31). The United States and Japan may have created multiple modernities by building university systems that reflect broader cultural differences in stable versus flexible social relationships.

## Retaining or growing?

Despite having roots in previous theorizing, the seed model has a basic difference from most of the ideas that have pushed back on the idea of convergence. The difference is that the seed model argues for the growth of cultural differences from their long-run historic baseline. Of the ideas that have pushed back against convergence, most argue that cultural differences are remaining or reviving.

Take Eisenstadt as an example. He wrote that his idea “goes against the view of the “classical” theories of modernization and of the convergence of industrial societies” (27). He clearly disagreed with the idea of convergence. But it was not clearly an argument about the growth of cultural differences. Instead, the primary argument was that societies can modernize in different ways.

Earlier, I pointed out how Ronald Inglehart argued that modernization pushes cultures in a “common direction” (5). Yet, he did not predict that cultures would converge. How can the effect of modernization be “common” but still fail to shrink differences?

He argued that cultures are going in the same direction but on “parallel trajectories” (5). Those parallel lines extend over time, allowing for predictable change without decreasing cultural differences. He doubted that “the forces of modernization will produce a homogenized world culture in the foreseeable future” (5).

Huntington seemed to suggest a different model—decline followed by revival. For example, he used words like *return* and *re-emerge* to describe differences (32). That suggests that cultures were converging but are now returning to their long-run differences.

In short, there is a common thread connecting researchers who are pushing back against the idea that modernization is erasing cultural differences. Yet many of these scholars argue that cultural differences are persisting or reviving, rather than growing from their historical size. Braudel was an exception (22). I think the scarcity of researchers arguing for real growth of cultural differences with modernization is a sign of how unexpected this pattern is.

## Modernization might amplify differences many people do not think of as culture

Up to this point, I have been using the word *culture*. My choice of words can easily make people think of countries, but I want to avoid that trap. I suspect the seed model could apply to group differences that people do not think of as cultural.

For example, psychologists often call gender an “individual difference” (33). But genders clearly share things like ways of talking, clothing styles, norms, and media. Genders are groups.

There is some evidence that gender differences are larger in more modernized countries (34). There is fierce debate about whether gender differences are social or biological, and my purpose here is not to settle that debate. What is important here is only whether modernization shrinks these group differences.

Economists analyzed data from 80,000 people using representative samples across 76 countries (34). They measured a range of decisions and preferences, such as risk-taking, patience, and altruism. Countries with higher GDP per capita had larger gender differences (*r* = 0.67, *P* < 0.001). Gender differences were among the largest in Canada and Sweden and the smallest in Pakistan and Ghana. This result is surprising given the greater legal equality between men and women in modernized countries.

The effect of genes on IQ offers an example more explicitly connected to biology. Twin studies consistently show that genes can explain a substantial portion of variation in IQ, yet genes explain more variation in wealthy communities than in poor communities (35). Genes for high IQ may give people preferences for reading and learning, but modernization affords kids the chance to express those preferences. Kids with the genetic potential for high IQs seem to be missing the nurturing environment they need to develop that intelligence.

## Four mechanisms for growing differences

The mechanism I laid out is that modernization gives people resources, and people can choose to use those resources in ways that express underlying differences. My explanation hinges on choice and expression, and there is some evidence that modernization increases choice. As a small example, one researcher compared how often American English books mentioned the word *obliged* versus *choose* from 1800 through 2000 (4). In the 1800s, *obliged* was about 7 times more common. By 2000, their positions had flipped. *Choose* was now about 7 times more common than *obliged*. This fits with the idea that choice is increasing with modernization, but we need more tests with different methods.

There are other possible mechanisms besides choice and expression. A second mechanism closely related to choice is selective migration. Planes, Internet, and global bank networks make it easier to choose to live somewhere else. People could choose where to live based on their cultural fit. For example, there is some evidence that Americans tend to feel out of place if they are liberal but live in a conservative community or vice versa (36). Those ideological misfits are more likely to move (36). Although migration involves choice, it is different from my simple model because modernization is not changing the same person over time. Instead, it could be increasing regional differences because people simply sort themselves into communities of similar people.

A third possible mechanism is awareness. Huntington argued that contact makes people hold more tightly to their cultural identities (32). Things like travel, trade, and media put people in more contact than before. “These increasing interactions intensify civilization consciousness and awareness of differences between civilizations,” according to Huntington (32). And because people are now more aware of their differences, it “invigorates differences or animosities stretching … back deep into history” (32).

The awareness mechanism makes sense with Brewer's psychological theory of optimal distinctiveness (37). The idea is that humans have competing needs to feel belonging and to feel distinct. Brewer argued that groups that are too large fail to meet people's basic need to feel distinct. This psychological need could push people to resist the forces of modernization, even if those are merely people's perceptions that they are becoming too similar to other groups.

A fourth mechanism is government and politics. Political parties could appeal to voters' need to feel distinct from others by emphasizing issues that divide social groups. For example, governments can “inflame religious, racial, ethnic and cultural divisions” to win votes or distract from their failings (38). Huntington argued that this type of cynical divisiveness is becoming more common: “Governments and groups will increasingly attempt to mobilize support by appealing to common religion and civilization identity” (32).

Power was a thread throughout Huntington's work. He believed cultural change depended heavily on political power. Part of his prediction that Western and non-Western cultures would diverge was based on “the passing of European empires and the eventual decline of US” power (24). Implicit in this view is that governments and political power drive cultural change.

This is obviously true in some cases. Governments sometimes intervene directly in cultural differences. Australia's programs to assimilate Aboriginal communities in the 1900s are just one example.

Scholars have also pointed to cases of government support that falls short of compulsion. In his book *China's New Confucianism: Politics and Everyday Life in a Changing Society*, Daniel Bell describes how the government funds Confucius Institutes to promote Chinese culture (39). He also points to the strong Confucian themes in the opening ceremony for the 2008 Beijing Olympics. Governments make decisions about how to spend money, and they can choose to spend that money in ways that reinforce cultural differences.

How can researchers test these mechanisms? The strongest evidence will span different methods and fields, by combining trends in polling data, election results, and lab studies. For example, new text databases (40–42) have come online documenting politicians' speeches across parties in Europe and the United States over time. Researchers could analyze whether these speeches appeal to identities and division over time with increases in markers of economic development, trade, and travel.

Of course, a downside of using observational data is that it is hard to prove causality or to test precise mechanisms. To do that, researchers could run lab studies to more precisely test mechanisms. For example, researchers could give participants (selective) evidence that their nation, city, or university is becoming more similar to other schools and see whether that triggers a motivation for distinctiveness. That information might also motivate a desire to move, which would fit with the selective migration mechanism.

## Where should we look for seeds?

If researchers generally assume modernization reduces cultural differences, we might miss the ways modernization seems to increase some types of cultural differences. My goal in this article is to bring attention to the that divergence and encourage research on it. If this call to action is successful, the seed model has many difficult questions to grapple with. Most critical are the questions of where these seeds come from and how they change. I outlined farming history as one source, but there are many plausible sources.

Huntington confidently planted a flag in religion (24). Religions played a large role in the map of world cultures he drew. For example, Hindu, Buddhist, and Orthodox were some of the categories he used.

Eisenstadt traced differences back to the “axial age” between 800 and 200 Bce (27, 43). The idea was that the era of Plato and Confucius was when several of the world's major cultures developed philosophies and religions left lasting influences. That changed the trajectory (the “axis”) of different civilizations.

One problem with the axial age idea is that written records become much more sparse going back further in history. That makes it difficult to know whether this era was the birth of those ideas or if it was a continuation of earlier patterns. For example, Confucius claimed to be continuing rituals from the Shang dynasty before him, but there was debate until recently whether the Shang dynasty ever existed, let alone whether Confucius was continuing earlier traditions faithfully or putting his own spin on it (44).

Other researchers have pointed elsewhere. The historian David Hackett Fischer argued that institutions are critical in influencing culture (45). The basic idea is that whichever cultural group set up institutions like schools and rules for government play an outsized role in the culture (46). One term for this is the “first effective settlement” (47). This helped Fischer explain why Pilgrims in Massachusetts had lasting cultural influence despite the fact that the descendants of English settlers were later far outnumbered by Irish immigrants.

The rice farming example here is a part of a larger bucket of ecological theories of culture [Talhelm and Oishi (48) provide a review of ecological theories]. The ecology makes some subsistence styles possible and others impossible or at least far less rewarding, such as farming in Greenland or fishing in Nevada. Researchers have also pointed to how ecological conditions shape diseases, which then shape cultural practices. For example, Harry Triandis argued that the tsetse fly shaped culture in some parts of Africa because they made raising cattle more difficult (49).

## When seeds and when radiation?

I should be clear that I do not expect the seed model to apply in every case. Instead, the question is when it applies. When is the seed model a better description of the effect of modernization, and when is the radiation model a better description? If the seed model is to become useful, it needs to be able to make useful predictions about when we should predict loss of diversity and when we should predict increased diversity.

One plausible dividing line is between behaviors that are directly tied to money versus more abstract preferences, attitudes, and values, which are less clearly tied to money. This could be why the effect of modernization seems to be so consistent when it comes to things that governments measure, like divorce, birth rates, and living alone. Money makes it possible to live alone. Separate bank accounts and higher wages make it easier for people to divorce an abusive husband or wife. To me, it makes sense to predict a similar effect of modernization across cultures in these cases.

But the link between money and the questions in the World Values Survey is less clear to me. People can believe in God with or without a bank account. People can agree with obedience or the importance of family whether they are getting richer or poorer. When we have enough data to run meta-analyses on studies of convergence and divergence, I would expect that the seed model applies more to attitudes and values and less to money-constrained behaviors like living alone.

Whether we find convergence or divergence could also depend on who we are comparing. In the World Values Survey, countries that are near each other seem to be converging in their values, while larger regions (like Europe and Africa) are diverging (15). There is a similar pattern within Europe. Values in the European Values Survey converged between 1992 and 2011 for countries in the European Union, but they diverged between the European Union and European countries outside the union (50).

The European Union finding links to the broader idea that political groups and trade flows help determine which cultures diverge and which cultures converge (1). The idea here is simple: “increased interaction among countries leads to cultural convergence” (but keep in mind that Huntington argued the opposite) (1). Researchers can test this using proxies for interaction, such as trade flows, travel, and interaction on social media.

At the same time, it is reasonable to expect that interaction can be unequal. One case is language. About 4% of words in Japanese are borrowed from English, whereas far less than 1% of English words are borrowed from Japanese (51, 52). I outline these estimates more in the Supplemental Material. We can think of cultures as having gravity (53). Cultures that are bigger may have more gravity than others, which pulls other cultures closer.

Sheer size (or size of the economy) could be a key to that gravity. For example, the United States has a bigger economy and population than Canada and Mexico. That would lead to the prediction that Canada and Mexico would become more similar to the United States over time than vice versa. The economic explanation is similar to what some researchers have called the “prestige bias,” which describes the tendency to learn from people who are successful (54).

## Separating the elements of modernization

Another important question for any model of modernization is to be clear about what modernization is. This is a deceptively simple question because it can seem obvious what modernization is. But there is room for disagreement, and the elements are tricky to separate. Table 1 gives a brief list of some of the central elements of modernization.

**Table 1.** A brief list of the elements of modernization.

Element	Description	Example measures
Economic development	Economic development is arguably the main element of modernization. Researchers often use GDP per capita as a proxy for development, but some researchers have argued that other proxies are better (7).	GDP per capita, income, food supply
Economic system	Industrialization is a commonly used descriptor of modernization. Inglehart argued for measuring the shift from resources (like mining and oil) and manufacturing to the service economy. Researchers have also measured the shift from a state-planned economy to private enterprise and integration into a market economy as markers of modernization.	Percent service sector economy, employment in private industry, market integration
Technology	Technology is another element of modernization, bringing things like air conditioning, running water, and smartphones.	Percentage of homes with Internet installed
Globalization	An important element is globalization, or social exchange. Some could argue that exchange should be considered an element of economic development because development and technology make that exchange possible. But some researchers write about globalization with a meaning that is not perfectly interchangeable with modernization (1).	Trade flows, foreign visitors and students
Urbanization	People moving to cities must shift away from traditional subsistence styles and toward market economies.	Percent living in urban areas, population density
Education	Education pushes people toward rationality and science (two other factors that some researchers could argue are elements of modernization).	Percent college educated, years of schooling

This table outlines some of the central elements of modernization. I do not intend the list to include every possible element. Instead, I hope that it serves as a useful reference point for researchers to test the different elements of modernization.

My goal in listing these elements is not to exhaust every possible element. People could argue that rationality, the loss of religion, and the decline of authority should be on this list (5, 10). Instead, my goal is to point out why separating the elements might be helpful for testing the seed model.

Simply put, the effect of modernization may depend on which element of modernization is changing. Education and urbanization could have different effects from globalization and economic development. Even an element like technology is a big bucket, with the potential for many different effects. It is worth asking whether these have distinct effects, rather than tacitly assuming they should all have the same effect because they can all be called “modernization.”

But this will be a challenge. The challenge is that modernization often comes with all of these elements packed together. Wealth helps countries fund education. But education probably builds wealth (55). If researchers just put all these elements into a regression soup, it is more statistical exercise than reality.

Yet there may be rare natural experiments that pull apart different mechanisms. One example is a policy in Norway that changed the mandatory education from 7 years to 9 years (56). Children born just days before the cutoff experienced the same national GDP but got 2 years less of school. Natural experiments like this could separate out at least some of the mechanisms behind modernization.

## Growth or convergence?

One deceptively simple question is what people mean by economic development. One version is simply growth. Another version is requiring convergence—less developed places need to shrink the gap with wealthy, mostly Western cultures.

Let's take the example of GDP per capita in Vietnam and the United Kingdom. The World Bank has estimates of their GDPs from 1990 to 2024, which overlaps with much of the World Values Survey analysis (Fig. 1). Vietnam had a higher growth rate (1,255%) than the United Kingdom (255%) (57). But the United Kingdom gained more dollars ($43,566) than Vietnam ($15,176). If researchers are looking for countries to converge in absolute GDP per capita over time (1), then Vietnam and the United Kingdom would be an example of the opposite. A reasonable person could look at that data and ask why anyone would expect Vietnam and the United Kingdom to become more similar.

Then again, that line of thought treats all dollars the same. A plausible alternative is that the first $1,000 or $10,000 are much more transformative than the change from $70,000 to $80,000 that the United States has experienced in the last 4 years (57). That would parallel the research on money and happiness, finding that money seems to make a bigger difference on the low end (58).

What's more, analyses have found that inequality between nations has decreased in recent decades (2, 59). In other words, even if the requirement is that countries are converging in income, that seems to have happened around the time of the World Values Survey analysis.

Beyond that, most other indicators of modernization in Table 1 seem to be converging over time. When the World Values Survey started, education and urbanization used to be much more unequal between wealthy, Western countries and developing, non-Western cultures. Since then, there has been convergence. Estimates of global interconnectedness (60) like foreign direct investment, international tourism, and even voice traffic, have increased too. I give a rundown of the convergence over time in the Supplemental Material.

## Seeds have hard shells but can still crack

Sunflower seeds and apple seeds have hard shells to protect them from being changed by the environment (such as radiation from the sun). But that does not mean change is impossible. We need studies to test what factors tend to change seeds and what factors just produce temporary changes.

I would also encourage researchers to consider the possibility of a time lag between modernization and cultural change. Cultures do not change on a dime. It takes time for structural changes to diffuse into people's mindsets.

Some studies have looked at a time lag between economic development and cultural differences (10, 14, 61). There is some evidence that changes in the US economy predict changes in behaviors like divorce and living alone more strongly five years later, rather than one year later (10). But the default is still to test GDP per capita in the same year as the cultural marker. In my own work, I have added analyses of time-lagged GDP only to face peer reviewers who asked me to remove them.

Childhood environments could be a place to look. If people tend to develop attitudes and values when they are growing up, the environment from their childhood might be a better predictor than the current environment. This idea fits with the finding that people tend to stream songs on Spotify that were popular at the time they were 13 years old for women and 14 years old for men (62).

## Retaining human agency

The seed model will also have to deal with the danger of essentializing cultures. The danger is that people see culture as so enduring that they ignore variation between individuals. Reading classic texts like the *Analects* of Confucianism or the *Vedas* of Hinduism and think they have such a powerful understanding of people in China or India that they do not need to ask people there now what they think.

This would be a grotesque extreme of the seed model. We must recognize that humans also have the capacity to question the seeds. Seeds are not fate.

But I think it would be wrong to think of seeds as incompatible with human agency. In one sense, the seed model highlights agency. It points out how people are active decision makers in how to use resources, not mere widgets on the conveyer belt of modernization.

## Text analysis could give us access to hundreds of years of study participants

There is a critical problem for researchers like me who want to test ideas about cultural change. We need more data, preferably longitudinal. The most obvious kind of data we can use is by simply asking people about their attitudes. But even impressive efforts like the World Values Survey only go back a handful of decades. Census data on markers like living alone often go back further but is often still limited to the 1900s (10).

Text analysis may be opening up many more centuries of data (63, 64). More and more text databases are becoming available. For example, researchers created *The Encyclopedia of Persian Stories* stretching back to the 900s, and that allowed researchers to quantify differences in romantic love over historical periods (59).

And just as databases are becoming available, new tools like large language models (LLMs) are giving researchers tools to analyze that text far more efficiently. Researchers have tried methods like asking ChatGPT to rate how much each text has themes of romantic love from 0 (*not at all*) to 9 (*a great deal*) (65). We must be careful not to sacrifice critical thought in this process. However, researchers have compared LLM coding with human coders and found that the two largely agree (65).

Another method is to create an LLM by feeding it a database of historical texts, say from Ancient Rome. Then researchers can ask the LLM to respond to questions as if it were a participant in a study (64). Of course, it would be foolish to stop running studies with human participants. But researchers can cross-check the validity of these estimates against other markers, such as benchmarking LLM ratings of satisfaction with government to historical records of revolts.

Methods like these can help us answer critical questions to understand how useful the seed model is. By looking back into history, we can learn what people were like. And by tracking these changes over time, we can learn how people have changed as modernization marches on.

## Supplementary Material

pgag021_Supplementary_Data

## Data Availability

This study did not obtain Institutional Review Board approval or consent from participants because the data came from publicly available surveys and Census data. Data and analysis syntax are available on the Open Science Framework: https://osf.io/38aqf/?view_only=c874f49e31154cdfb1da7d33c4fd1cc4
