# Supplementary material for: Is modernization widening cultural differences?
Source: PNAS Nexus. 2026 Mar 10;5(3):pgag021. doi: 10.1093/pnasnexus/pgag021 (PMC12973498; doi:10.1093/pnasnexus/pgag021)
Supplement: pgag021_Supplementary_Data [file pgag021_supplementary_data.pdf]

Supplemental Materials

In the section "Growth or Convergence," I talked about different versions of modernization theory. Some versions require countries to converge over time in indicators like GDP and urbanization. Some do not. In this section, I cover a few of the mechanisms people have proposed for cultural convergence with modernization. For each mechanism, I ask whether convergence might be required. And where possible, I offer statistics on whether there has been convergence between wealthy, Western regions and developing, non-Western regions during the time period of the World Values Survey analysis in the paper on value divergence (1).

1. Civilizational Progress

Some versions of modernization don't require convergence in indicators like GDP. For example, Fukuyama's version is that cultures are learning from each other about the most optimal form of government and economy (2). By that account, cultures should be converging, regardless of GDP.

2. Globalization

Increasing interconnection is one element that people often mention. For example, one scholar pointed out how Starbucks can make it so that:

> your morning cappuccino is the same no matter whether you are sipping it in Tokyo, New York, Bangkok or Buenos Aires...Select any global brand from Coca Cola to Facebook and the chances are you will see or feel their presence in most countries around the world. It is easy to see this homogenization in terms of loss of diversity, identity or the westernization of society (3).

The Swiss Economic Institute calculates the index using diverse indicators of interconnection, such as international trade, tourism, and even voice traffic (essentially, people making phone calls around the world). The value divergence paper used this index in some analyses (1). I graphed out the Globalization Index from 1980 to 2020 (Figure S1). This index shows interconnection has increased during the World Values Survey time period, in line with the Starbucks observation (3). That could lead to the prediction that cultural differences should be shrinking.

Figure S1

Globalization Index Increased from 1980 to 2020

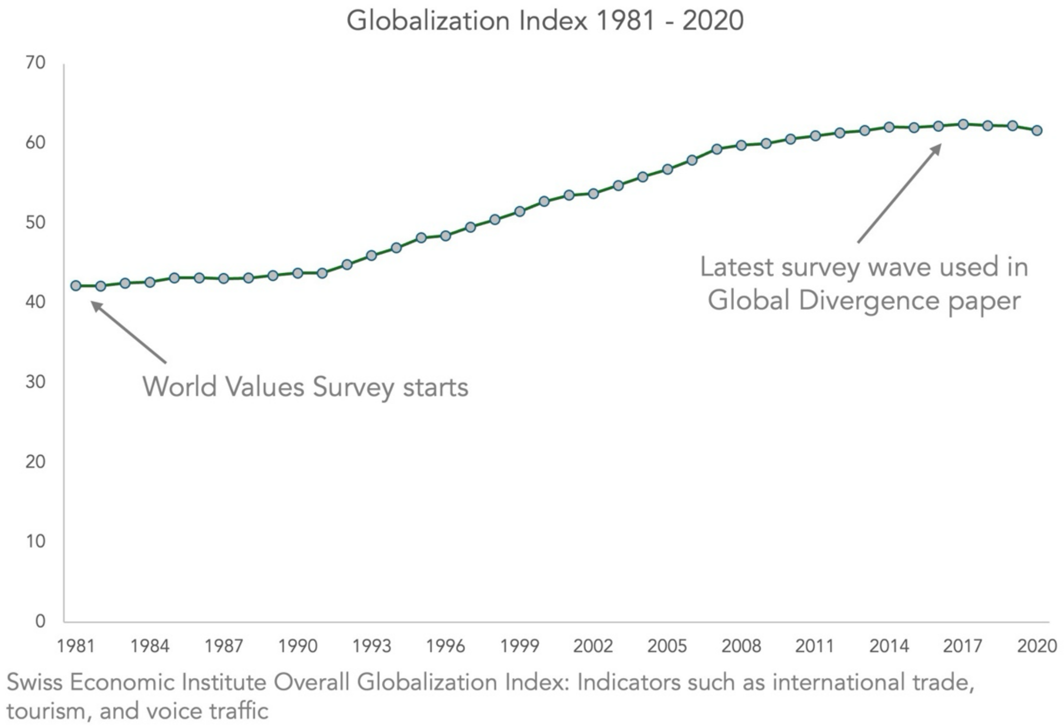

Note: The Swiss Economic Institute calculates its Overall Globalization Index based on indicators across 12 domains, such as financial globalization (such as foreign direct investment), interpersonal globalization (such as international tourism and migration), and political globalization (such as the number of international NGOs). This index of globalization has increased from the 1980s through the 2000s.

3. Urbanization

I compared urbanization rates across continents over time using the World Bank's data explorer. During the years of the World Values Survey analyzed in the global divergence paper (1981-2017), urbanization increased around the world (Figure S2). Looking at the rates of change, there was convergence between the highly urbanized regions and the less-urbanized regions. The less-urbanized regions had higher growth rates (as a percentage of baseline) and higher absolute changes (Figure S3). Based on this indicator of modernization, major world regions have seen convergence, and therefore it would be logical to expect cultural convergence.

I should point out one weakness is that the World Bank's data explorer tool doesn't have world regions that map exactly onto the regions in the global divergence paper (1). For example, the World Bank has data for the European Union, but not the European continent. However, the pattern of convergence seems consistent.

Figure S2

The Percentage of The Population Living in Cities from 1981 to 2017

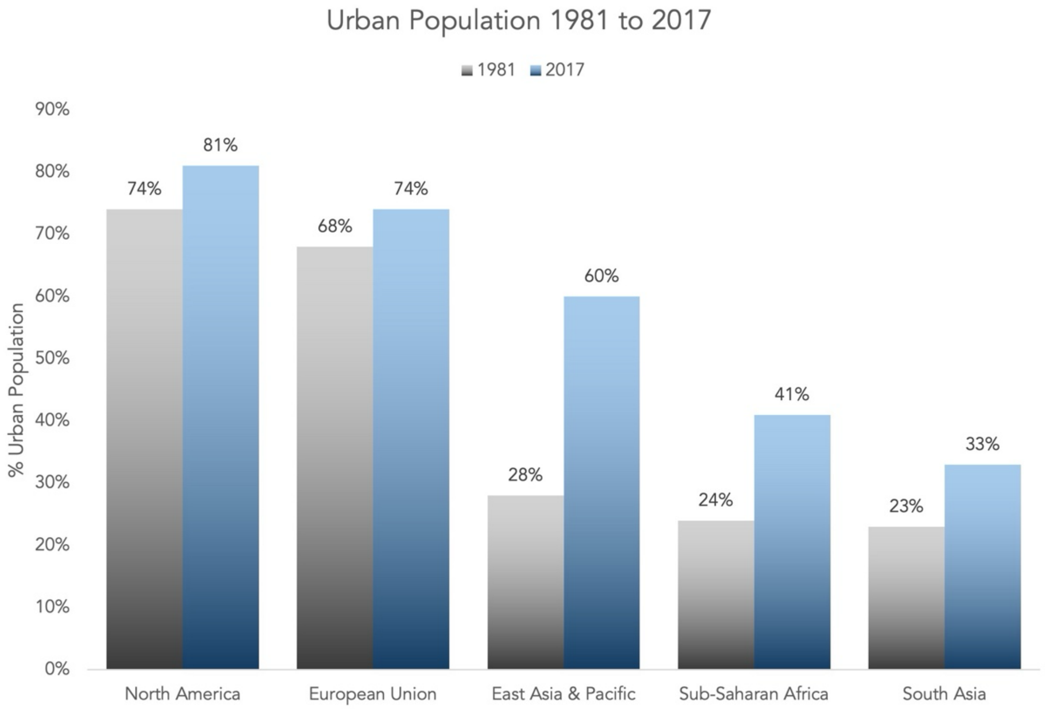

Note: This graph uses urbanization rates from the World Bank's data explorer from 1981 to 2017. This time period maps onto the years of the global value divergence study (1). In 1981, there was a large gap in urbanization between Western and non-Western regions. By 2017, that gap shrank.

Figure S3

Changes in Urbanization from 1981 to 2017

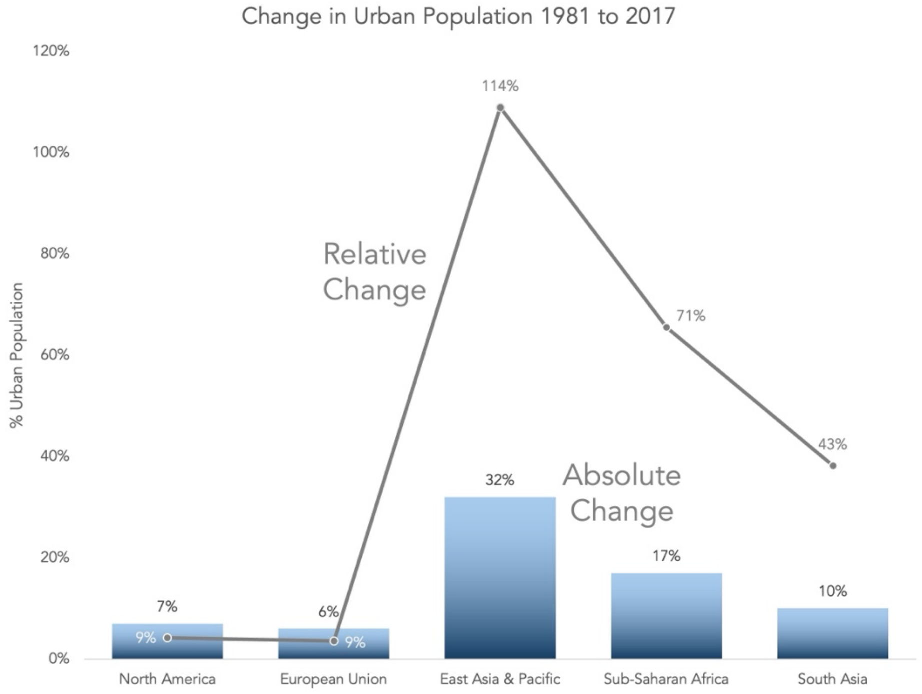

Note: This graph illustrates the rates of change in urbanization from the previous figure. The line illustrates relative change in urbanization (percentage of baseline), and the bars illustrate the absolute change. The three non-Western regions experienced higher rates of absolute and relative growth than the two Western regions.

4. Education

I analyzed data on education across regions from Our World in Data from 1980 to 2020. This data is only available every five years, so it does not line up as precisely to the World Values Survey data. The metric is the average years of schooling among people aged 15-64 years old.

Each world region increased in education across these 40 years (Figure S4). However, the less-developed regions increased more than developed regions. South America, Asia, and Africa all increased over 90%. Europe and North America increased less than 50% (Figure S5). Looking at absolute change, Europe gained slightly more than Africa, but all of the other comparisons showed larger absolute growth in places that had less average education to begin with. Thus, if people look at education levels as a marker of modernization, it would be logical to predict cultural convergence.

Figure S4

Years of Schooling Across World Regions from 1980 to 2020

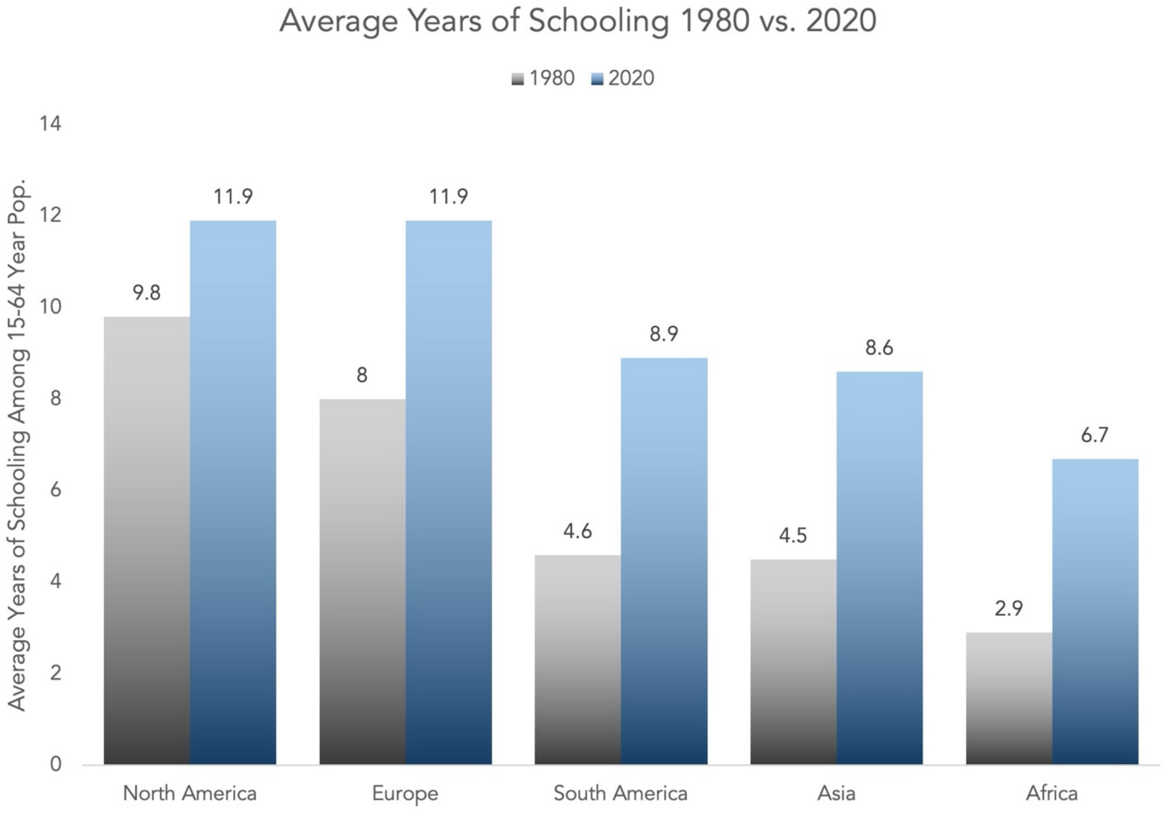

Note: This figure plots the average years of schooling across world regions from 1980 to 2020. These figures are for the population aged 15 to 64 years old. The data comes from Our World in Data.

Figure S5

Change in Years of Schooling Across World Regions from 1980 to 2020

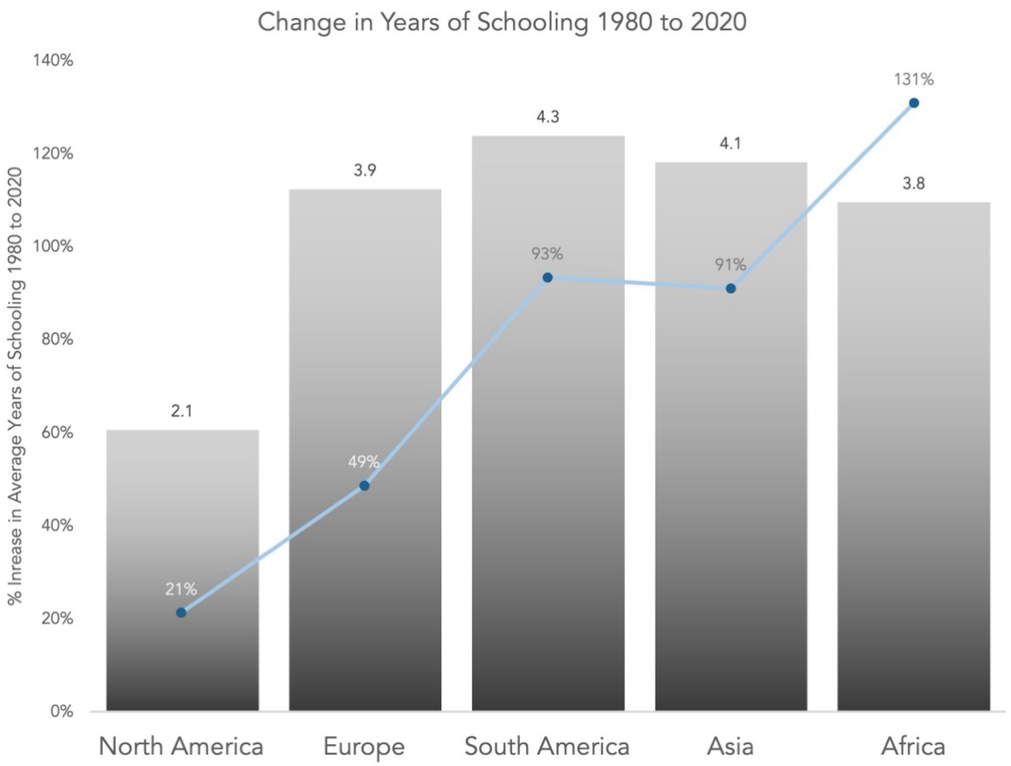

Note: This figure plots the change in education levels from 1980 to 2020 both as a percentage from baseline (the line) and in the absolute number of years (gray bars). Overall, the trends show convergence between developed, Western regions and developing non-Western regions (although there Africa outpaced Europe in relative terms but not absolute terms).

5. GDP growth rates

Another way to predict convergence is by looking at growth rates in economic development indicators like income and GDP per capita. If GDP is growing at a higher rate in non-Western, less-wealthy places, then this would be a logical reason to predict cultural convergence.

A World Bank report summarized growth rates from 1990 to 2019, which covers most of the time of the World Values Survey data (4). Income per capita grew at higher rates in lower-income countries (Figure S6). Of course, for any of the metrics here, there is still room for more nuanced theories of time-lagged changes and non-linear effects (such as, 5). In other words, this analysis is not the final word on the topic. But growth does seem to be higher in less-developed regions.

Figure S6

Income per Capita Growth Rates Were Higher in Low-Income Countries (1990-2019)

A. 1990 – 2019 (Historical)

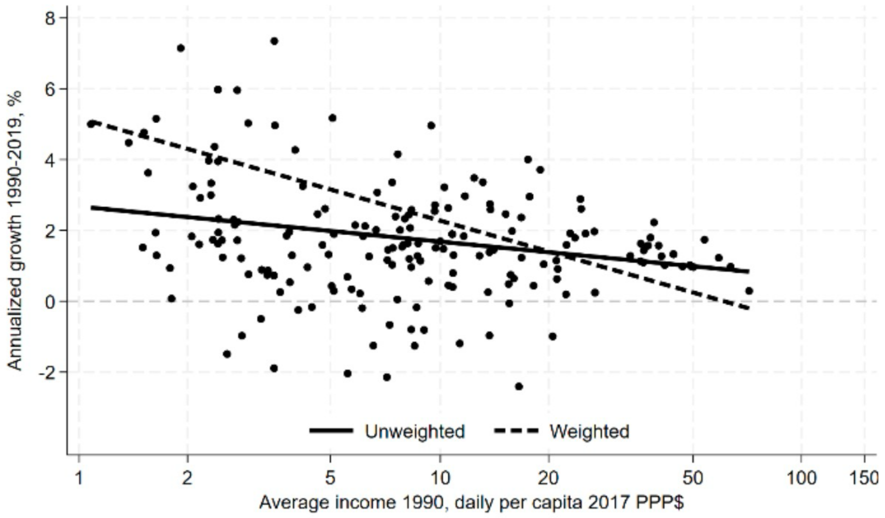

Note: This graph comes from a World Bank report (4). The solid line treats every country as an equivalent data point. The dashed line weights countries by their population size (for example, weighting China and India more heavily than Sri Lanka).

6. GDP in absolute terms

I saved this for last because I think it's the most complicated question. Put another way, I think this is probably the easiest place to critique the convergence expectation.

The skeptic case: People could look at the graph of growth rates (Figure S6) and argue that, sure, wealthy countries are growing more slowly, but they're gaining more actual dollars. In the main text, I gave the example of GDP per capita in Vietnam and the UK from 1990 to 2024. Vietnam's GDP growth rate (1,255%) was higher than the UK's (255%). But the UK gained more absolute dollars ($43,566) than Vietnam ($15,176).

I would respect any researcher who argues that that should lead to cultural divergence, rather than convergence. It's a reasonable position to take.

However, I think a key factor is that people's predictions depend on whether people think that effects of economic development on cultural change are linear or non-linear. If it's linear, then we should not predict convergence. But the effect of money may be non-linear. Dollars might matter more when people have very little to start with. That seems to be the case with money and happiness (6). If that's the case, then people might predict convergence between Vietnam and the UK.

I think cases of convergence in absolute dollars are harder to find than for growth rates. There are some cases, mostly in the Middle East (such as Saudi Arabia, UAE, Qatar, and Kuwait) and East Asia (such as Singapore, Hong Kong, and Japan).

The case for convergence: Some analyses suggest that there has been convergence in wealth around the time period of the World Values Survey. One data point is the between-country Gini index (Figure S7). The World Bank analysis shows a drop from 1990 to 2019 (4).

Figure S7

The Gini Index of Inequality Between Countries

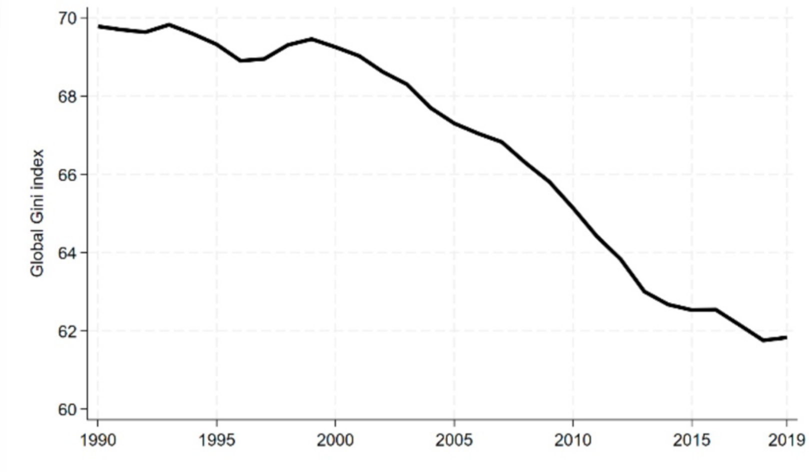

Note: This graph from a World Bank report illustrates the Gini index of inequality between countries (4).

Our World in Data has a stark figure that illustrates a similar pattern from 1975 to 2015 (Figure S8). Daily income per capita was essentially bimodal in 1975. There was a large lump on the low end and a large lump on the high end, which was mostly Europe and North America. But by 2015, the two lumps converged. It looks a lot more like a normal distribution now. Less-developed areas gained ground on rich areas, which would lead to a prediction of convergence, even under the criterion of absolute dollar amounts.

Figure S8

The Income Distribution Around the World Has Grown More Equal from 1975 to 2015

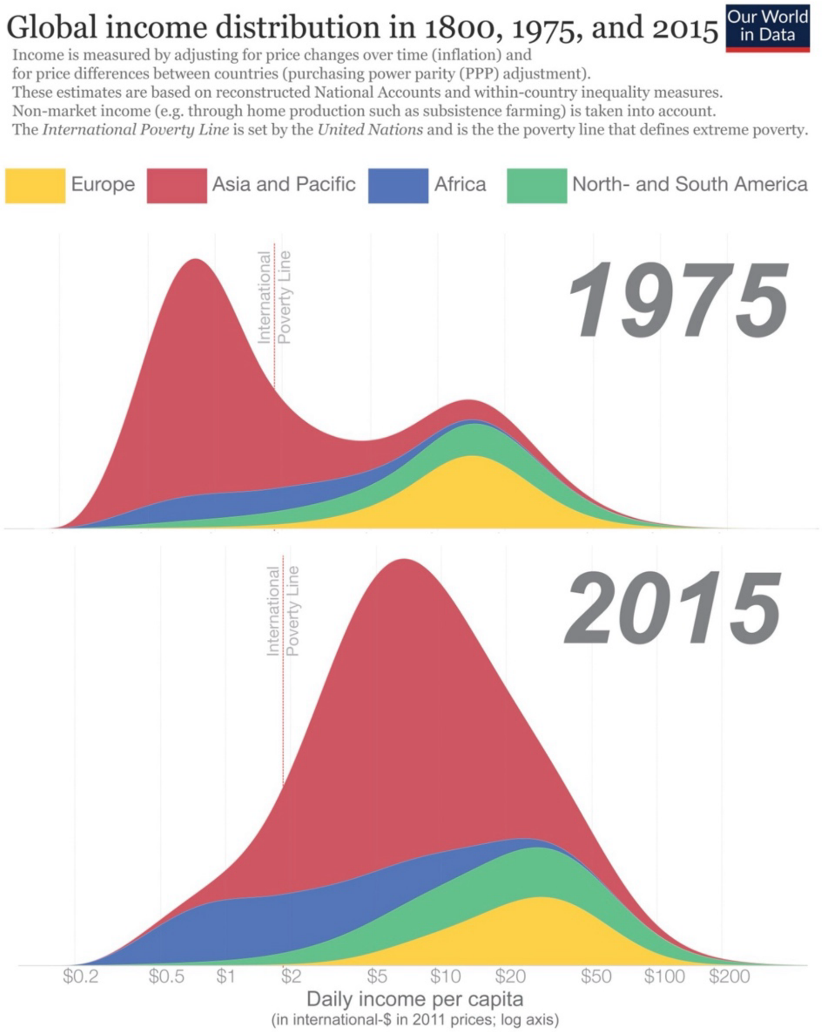

Note: This figure from Our World in Data illustrates income per capita across the world, taking into account purchasing power parity, inflation, and the value of subsistence activities, such as farming. This figure is free to reproduce under a creative commons license (CC BY).

However, I still acknowledge that cases of convergence in absolute dollars are harder to find than for growth rates, education, and urbanization. The difficulty of finding convergence in absolute terms is one reason why the rice-wheat comparison in China is so intriguing. The two regions started out pretty close in 1982:

Rice 1982 = 708 Yuan per capita
Wheat 1982 = 613 Yuan per capita

If anything, rice areas started out a bit wealthier, which would lead to the prediction that they should have higher divorce rates and more people living alone. The data trends in the opposite direction, although I think the difference in GDP is negligible.

Through 2020, rice areas experienced far more growth. Rice areas grew 11,755%, and wheat areas grew 10,139%. That's interesting because it means rice areas grew more than wheat areas **both** in absolute numbers and percentages.

In fairness, it doesn't show convergence. It shows the opposite—an area that started out more collectivistic to begin with growing faster than an area that started out less collectivistic. But if the idea is that economic growth causes individualism, it would be logical to hypothesize that rice areas should see more growth in individualism than wheat areas.

In sum, I think the bulk of the mechanisms of modernization have shown convergence over the last 40 years or so. Metrics of interconnectedness have increased (Figure S1). These are logical reasons to predict cultural convergence. However, cases of convergence in absolute dollars are rarer and more valuable for research on the question of cultural convergence.

Supplemental References

1. J. C. Jackson, D. Medvedev, Worldwide divergence of values. *Nat. Commun.* **15**, 2650 (2024).

2. F. Fukuyama, *The end of history and the last man* (Free Press, 1992).

3. M. Pagel, Does globalization mean we will become one culture? *BBC* (2014).

4. D. G. Mahler, N. Yonzan, C. Lakner, "The impact of COVID-19 on global inequality and poverty" (World Bank, 2022).

5. M. Myrskylä, H.-P. Kohler, F. C. Billari, Advances in development reverse fertility declines. *Nature* **460**, 741–743 (2009).

6. S. Oishi, *The psychological wealth of nations: Do happy people make a happy society?* (Wiley-Blackwell, 2011).

7. S. P. Huntington, *The clash of civilizations and the remaking of world order* (Simon and Schuster, 1996).
